# The Role of Immunotherapy in MMR-Deficient Endometrial Carcinoma: State of the Art and Future Perspectives

**DOI:** 10.3390/jcm13237041

**Published:** 2024-11-21

**Authors:** Matteo Marchetti, Jacopo Ferrari, Tommaso Vezzaro, Laura Masatti, Giulia Tasca, Tiziano Maggino, Roberto Tozzi, Carlo Saccardi, Marco Noventa, Giulia Spagnol

**Affiliations:** 1 Unit of Gynecology and Obstetrics, Department of Women and Children’s Health, University of Padua, 35100 Padua, Italy; matteo.marchetti@unipd.it (M.M.); tiziano.maggino@gmail.com (T.M.);; 2 Department of Biology, University of Padua, 35100 Padua, Italy; laura.masatti@unipd.it; 3 Medical Oncology 2 Unit, Veneto Institute of Oncology IOV—IRCCS, 35128 Padua, Italy

**Keywords:** endometrial cancer, mismatch repair deficiency, MMRd, immune checkpoint inhibitors, targeted therapy, molecular profile

## Abstract

This study provides a comprehensive overview of the role of immunotherapy in the treatment of mismatch repair-deficient (MMRd) endometrial carcinomas. Immunotherapy has emerged as a transformative approach in the treatment of MMRd due to the high mutation rate and subsequent PD-1/PD-L1 overexpression seen in these tumors. This review analyzes the current landscape of existing randomized clinical trials, highlighting the efficacy of immune checkpoint inhibitors (ICIs) like pembrolizumab, avelumab, and dostarlimab. Additionally, the focus extends to the potential of combined therapeutic strategies, such as the integration of ICIs with targeted agents, while also exploring the application of immunotherapy in non-traditional settings beyond advanced or recurrent disease. This includes emerging roles in the adjuvant and neoadjuvant contexts to prevent recurrence and target early-stage disease. These findings underscore the importance of tailoring treatments based on the molecular characteristics of each tumor and paving the way for future advancements in the field of gynecologic oncology. Despite promising results, this article acknowledges the necessity of further research to refine patient selection criteria and explore combination strategies that can overcome resistance mechanisms.

## 1. Introduction

Endometrial cancer (EC) is one of the most prevalent gynecological malignancies in developed countries, presenting significant clinical challenges due to its diverse histopathological and molecular characteristics [1]. Recent advancements in the field, particularly the integration of immunotherapy, have shown promising results for specific molecular subtypes, such as microsatellite instability-high (MSI-H) and mismatch repair-deficient (MMRd) tumors [2]. Understanding the evolution of these treatment strategies requires an examination of the molecular classification systems for endometrial cancer, notably, those established by The Cancer Genome Atlas (TCGA) and the Proactive Molecular Risk Assessment (ProMisE) initiative [3,4].

The TCGA project has been instrumental in enhancing our understanding of the molecular landscape of endometrial cancer. Through the analysis of genomic, transcriptomic, and proteomic profiles from over 370 endometrial carcinoma samples, TCGA identified four distinct molecular subtypes: POLE-ultramutated, MSI-H, copy-number low, and copy-number high. Each of these subtypes has unique genetic and molecular characteristics that influence the prognosis and therapeutic responses:

▪ POLE-ultramutated: These tumors exhibit mutations in the POLE gene, leading to an exceptionally high mutation rate. This results in a high neoantigen load, making these tumors highly immunogenic and generally responsive to immunotherapy, with a favorable prognosis.
▪ MSI-H: Tumors in this category result from deficiencies in the mismatch repair system, leading to high levels of microsatellite instability and a high mutational burden. This makes them excellent candidates for immunotherapy due to their enhanced immune system visibility.
▪ Copy-number low: These tumors are often hormone receptor-positive with fewer genetic alterations, generally following a more indolent clinical course compared to other subtypes.
▪ Copy-number high: Associated with serous histology and TP53 mutations, these tumors have numerous chromosomal alterations and a poor prognosis.

Building on TCGA’s findings, the ProMisE classification system offered a clinically applicable framework for classifying endometrial cancers using surrogate markers that align with the TCGA subtypes. ProMisE stratifies patients into four molecular subgroups: mismatch repair-deficient (MMRd), accounting for 25–30% of ECs; POLE-ultramutated (POLEmut) for 5–10%; p53-abnormal (p53abn) for 10–15%; and no specific molecular profile (NSMP) for 35–45%. This approach facilitates more personalized treatment strategies, enhancing the ability to predict outcomes and tailor therapies [4]. For instance, POLE-ultramutated tumors are often associated with favorable outcomes and, as well as MMRd tumors, tend to respond well to immunotherapy, while p53-abnormal tumors are characterized by a more aggressive course and poorer prognosis. Studies indicate that MMRd EC demonstrated a 5-year disease-specific survival (DSS) of approximately 80–85% and a progression-free survival (PFS) of around 65–70%, positioning them with an intermediate prognosis between that of the previous two groups [4]. The NSMP subgroup, representing a heterogeneous group of tumors, presents management challenges due to a lack of specific molecular targets, emphasizing the need for ongoing research to identify novel biomarkers and therapeutic approaches [5,6].

Overall, the integration of molecular features into endometrial cancer management represents a major advancement in the field, promoting personalized medicine. This review aims to provide a comprehensive overview of the current uses of immunotherapy in MMRd endometrial cancer, detailing the pathogenic basis for PD-1 and PD-L1 overexpression, the mechanisms behind the success of immunotherapy, ongoing clinical trials, and future perspectives.

## 2. Background

### 2.1. Immunogenicity and Pathogenic Basis of PD-1 and PD-L1 Overexpression in MMRd Endometrial Cancer

The immunogenic nature of MMRd tumors is attributed to their high mutational burden [7]. In MMRd tumors, the loss of function of key MMR genes like MLH1, MSH2, MSH6, and PMS2 results in the accumulation of DNA replication errors, leading to a plethora of neoantigens. These neoantigens are recognized as foreign by the immune system, prompting a robust immune response [8]. Similarly, POLE-ultramutated tumors exhibit ultra-high mutation rates due to defects in the POLE gene, generating a substantial neoantigen load that can be targeted by the immune system [9]. Despite their high immunogenicity, the clinical application of immunotherapy in POLE-ultramutated tumors is less immediate compared to that of MMRd tumors. This is largely due to the intrinsically favorable prognosis of POLE-ultramutated tumors, which often respond well to standard treatments [10]. As a result, the urgency to develop alternative therapeutic strategies like immunotherapy is lower. However, the potential for combining immunotherapy with standard treatments to further enhance outcomes remains an area of active research.

**Figure 1:**
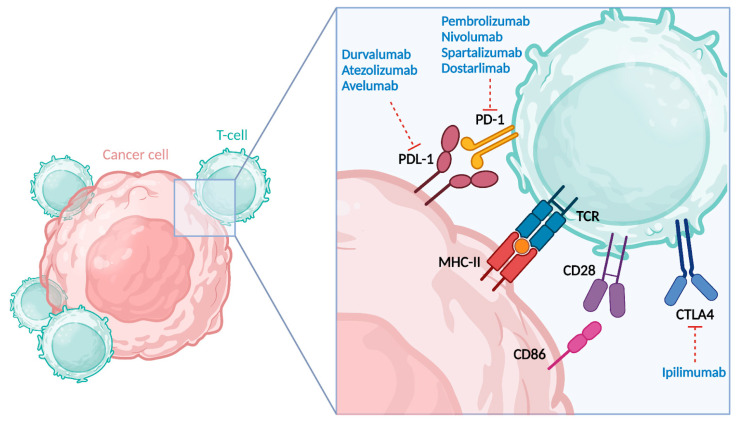
Interaction between a T cell and a cancer cell, highlighting key receptor–ligand pairs involved in immune checkpoint regulation. The figure emphasizes the interaction between the PD-1 receptor on the T cell and its ligand PDL-1 on the cancer cell, as well as the CTLA-4 receptor on the T cell. Both the PD-1/PD-L1 and CTLA-4 pathways are targeted by immune checkpoint inhibitors (ICIs), which help to reactivate the immune system’s ability to recognize and attack cancer cells [12]. *Created with BioRender.com*.

As previously mentioned, the abnormal production of aberrant proteins due to faulty DNA repair mechanisms is recognized by the immune system as foreign, triggering a robust immune response. However, tumor cells evade this immune surveillance by upregulating immune checkpoint proteins such as PD-1 and PD-L1, which inhibit the activity of T cells, thus allowing the tumor cells to survive and proliferate. Indeed, the interaction between PD-1 on T cells and PD-L1 on tumor cells inhibits T cell activation and allows the tumor to escape immune destruction. This mechanism of immune evasion underscores the rationale for using immunotherapy to block these inhibitory pathways and reactivate the immune system against the tumor [11]. Immune checkpoint inhibitors (ICIs) work by blocking the interactions between PD-1 on T cells and PD-L1 on tumor cells, as schematized in Figure 1.

### 2.2. Anti-PD-1 and Anti-PD-L1 Agents

Anti-PD-1 therapies function by targeting the PD-1 receptor on T cells, preventing its interaction with the ligands PD-L1 and PD-L2, which are often overexpressed on the surface of tumor cells. By blocking this pathway, these therapies disrupt the inhibitory signals that would otherwise deactivate the T cells [13]. This inhibition allows the T cells to remain activated, leading to a more robust immune response against cancer cells. Consequently, T cell-mediated cytotoxicity is enhanced, improving the ability of the immune system to detect and destroy malignant cells. Examples of such agents include Pembrolizumab, Dostarlimab, and Nivolumab, which have shown efficacy across several types of cancer, including melanoma, non-small cell lung cancer, and endometrial carcinoma [12,14,15].

Similarly, anti-PD-L1 therapies target the programmed death-ligand 1 (PD-L1), which is commonly expressed not only on tumor cells but also on certain immune cells, such as macrophages. By preventing the interaction between PD-L1 and the PD-1 receptor on T cells, these therapies inhibit the suppression of immune activity, ensuring that T cells remain active and engaged in attacking the tumor. This leads to a more effective immune response, reducing tumor growth and potentially improving patient outcomes. Examples of anti-PD-L1 agents include Atezolizumab, Durvalumab, and Avelumab, which have been approved for treating cancers like urothelial carcinoma, triple-negative breast cancer, and non-small cell lung cancer [16,17,18].

### 2.3. Anti-CTLA-4

Physiologically, the PD-1/PD-L1-mediated inhibitory mechanism is essential for maintaining immune tolerance and preventing autoimmune responses. Another key regulatory mechanism involves cytotoxic T-lymphocyte-associated antigen 4 (CTLA-4), primarily expressed in secondary lymphoid organs. CTLA-4 acts as a competitive inhibitor for the B7 ligand, which is expressed on antigen-presenting cells (APCs). This ligand, by binding to its co-stimulatory receptor, CD28, on T cells, promotes immune activation through the required two-signal process [19]. Over the years, CTLA-4 inhibitors, such as ipilimumab, have been developed to block this protein and enhance the immune response against cancer cells. While the use of CTLA-4 inhibitors is not yet as established in the treatment of endometrial cancer as it is for other cancers, such as Ipilimumab in melanoma or lung carcinoma, combining these agents with PD-1/PD-L1 inhibitors may represent a promising strategy to improve therapeutic efficacy in endometrial cancer [20].

## 3. Clinical Evidence and Current Indications—Advanced and Recurrent Disease

### Randomized Clinical Trials on Immunotherapy

A plethora of studies in the literature nowadays demonstrate the efficacy of immunotherapy, both as monotherapy and in combination with cytotoxic agents, in the treatment of advanced-stage (FIGO [International Federation of Gynaecology and Obstetrics] stage III and IV) and first-line recurrent MMRd endometrial carcinoma. Data published from significant studies, such as KEYNOTE-158, which showed the efficacy of pembrolizumab in patients with advanced MSI-H/MMRd endometrial carcinoma, and the ongoing phase Ib GARNET study [NCT02715284], which evaluated the efficacy and safety of dostarlimab in patients with MMRd endometrial carcinoma, led the EMA and FDA to approve the use of immunotherapy in these settings [13,21]. Consequently, ICIs were included in the 2023 ESMO Clinical Practice Guideline for diagnosis, treatment, and follow-up of endometrial cancer, which considered the use of ICIs as monotherapy in patients with MSI-H/MMRd EC after platinum-based therapy failure [22].

A recent meta-analysis reviewed the data from five randomized clinical trials on the use of immunotherapy in the setting of advanced stages and first recurrent endometrial carcinoma, excluding all studies where this therapy was offered as a second or subsequent line of treatment [23]. In the analysis conducted on a total of 2456 patients with endometrial cancer, it was observed that the use of ICIs in combination with chemotherapy was associated with a significant improvement in PFS compared to chemotherapy alone. A greater benefit was noted in patients with MMRd tumors. Specifically, the meta-analysis reported a pooled hazard ratio (HR) of 0.34 (95% CI, 0.27–0.44; *p* < 0.001), indicating a significant improvement in outcomes, and this benefit was consistently observed across different classes of immune checkpoint inhibitors. The authors also found that the type of ICI used does not significantly affect the effectiveness of immunotherapy. Moreover, the addition of ICIs to chemotherapy also demonstrated benefits in the mismatch repair proficient (MMRp) population.

Given the importance and significance of the studies considered in this meta-analysis, they are individually reported and analyzed below. Table 1 presents the main randomized studies on immunotherapy in EC along with their respective outcomes, while Table 2 provides a detailed summary of the key characteristics of these studies.

The MITO END-3 study by Pignata et al. was a phase III trial comparing the use of immunotherapy in combination with chemotherapy in 125 patients with histologically confirmed advanced (FIGO stage III–IV) or recurrent endometrial cancer [24]. The participants were randomly assigned to receive either carboplatin and paclitaxel intravenously every 3 weeks for six to eight cycles vs. carboplatin and paclitaxel with the addition of avelumab (10 mg/kg intravenously) every 3 weeks (experimental arm), followed by avelumab as single-agent maintenance therapy every 2 weeks after the completion of chemotherapy (until disease progression or unacceptable toxicity). No significant differences were observed between the two groups in the overall sample, with a median progression-free survival of 9.9 months (95% CI 6.7–12.1) in the standard group and 9.6 months (95% CI 7.2–17.7) in the experimental group. A subanalysis based on the MMR status revealed a significant interaction between the treatment effect and MMR status for both PFS (*p* = 0.015) and OS (*p* = 0.029). Specifically, in the MMRd subgroup, the PFS rates at 24 months were 50% (range 29–68) in the experimental group versus 13% (range 4–30) in the standard group. Regarding the overall survival, the 24-month rate was 76% (range 51–89) in the experimental group compared to 55% (range 33–73) in the standard group. 

In the RUBY trial, Mirza et al. randomized 494 patients with primary advanced stage (III or IV), or first recurrent endometrial cancer, to receive carboplatin and paclitaxel in combination with dostarlimab (500 mg) in the experimental arm or placebo in the control arm, every 3 weeks for six cycles, followed, respectively, by dostarlimab (1000 mg) or placebo every 6 weeks for up to 3 years [25]. The authors reported a PFS at 24 months of 36.1% (95% CI, 29.3 to 42.9) in the dostarlimab arm compared to 18.1% (95% CI, 13.0 to 23.9) in the placebo group (HR, 0.64; 95% CI, 0.51 to 0.80; *p* < 0.001). The OS at 24 months was 71.3% (95% CI, 64.5 to 77.1) with dostarlimab and 56.0% (95% CI, 48.9 to 62.5) with placebo (HR for death, 0.64; 95% CI, 0.46 to 0.87). Among the 118 patients with MMRd or MSI-H tumors, the estimated PFS at 24 months was 61.4% (95% confidence interval [CI], 46.3 to 73.4) in the dostarlimab arm compared to 15.7% (95% CI, 7.2 to 27.0) in the control arm (HR for progression or death, 0.28; 95% CI, 0.16 to 0.50; *p* < 0.001).

In the randomized NRG GY018 trial, Eskander et al. included 816 patients with advanced (stage III or IV) or recurrent endometrial cancer who were assigned to receive pembrolizumab vs. placebo in combination with paclitaxel and carboplatin [26]. The patients were stratified into two cohorts based on their mismatch repair status: one with MMRd disease and another with MMRp disease. It should be noted that this is the only study among those cited that does not have PFS in the intention-to-treat (ITT) population as the primary outcome (thus assessing the effect of immunotherapy on MMRd ECs only through a subgroup analysis). Instead, the study preemptively stratifies the population into two cohorts based on the MMR status. The estimated PFS at 12 months in the MMRd cohort was 74% in the pembrolizumab group compared to 38% in the placebo group (HR for progression or death, 0.30; 95% CI, 0.19 to 0.48; *p* < 0.001), representing a 70% reduction in the relative risk. In the MMRp cohort, the median progression-free survival was 13.1 months with pembrolizumab versus 8.7 months with placebo (HR, 0.54; 95% CI, 0.41 to 0.71; *p* < 0.001).

In the DUO-E trial, Westin et al. randomized 699 patients with newly diagnosed advanced or recurrent endometrial cancer to one of three treatment arms: carboplatin/paclitaxel plus placebo followed by placebo maintenance (control arm); carboplatin/paclitaxel plus durvalumab followed by maintenance with durvalumab and placebo (durvalumab arm); or carboplatin/paclitaxel plus durvalumab followed by maintenance with durvalumab and olaparib (durvalumab plus olaparib arm) up to a maximum of 24 months [27]. Statistically significant PFS benefits were observed in both the durvalumab arm (HR 0.71 [95% CI, 0.57 to 0.89]; *p* = 0.003) and the durvalumab plus olaparib arm (HR, 0.55 [95% CI, 0.43 to 0.69]; *p* < 0.0001) compared to the control. Analyses based on the MMR status revealed that, similarly to previously reported studies, in the MMRd subgroup, a clinically meaningful benefit was observed with both durvalumab compared to the control (HR, 0.42 [95% CI, 0.22 to 0.80]) and durvalumab plus olaparib compared to the control (HR, 0.41 [95% CI, 0.21 to 0.75]). However, the results in the MMRp subgroup were markedly different: a clinically significant benefit was seen with durvalumab compared to the control (HR, 0.77 [95% CI, 0.60 to 0.97]), and the addition of olaparib to maintenance therapy with durvalumab indicated further enhancement (HR compared to the control, 0.57 [95% CI, 0.44 to 0.73]). Thus, in this patient subgroup, while durvalumab showed benefits, the addition of olaparib provided further improvement, with greater benefit observed in the MMRp patients compared to the MMRd patients.

In the AtTEnd trial, Colombo et al. randomly (2:1) assigned 551 patients with advanced or recurrent endometrial carcinoma or carcinosarcoma to receive either intravenous atezolizumab (1200 mg) or placebo in combination with chemotherapy (carboplatin and paclitaxel) for 6–8 cycles, continuing until disease progression [28]. The median PFS for the patients on atezolizumab was 10.1 months (95% confidence interval [CI], 9.5–12.3), compared to 8.9 months (95% CI, 8.1–9.6) for those receiving placebo. This yielded a hazard ratio (HR) of 0.74 (95% CI, 0.61–0.91; *p* = 0.022), indicating a statistically significant improvement in PFS for the atezolizumab group. For the OS, the median was 38.7 months (95% CI, 30.6–not estimable) in the atezolizumab arm, versus 30.2 months (95% CI, 25.0–37.2) in the placebo arm. The hazard ratio for the OS was 0.82 (95% CI, 0.63–1.07) with a log-rank *p*-value of 0.048, suggesting a trend toward improved survival with atezolizumab, although this result approaches statistical significance in this interim analysis, with data maturity at 43%. It is important to note that 24.3% of patients in the placebo arm received subsequent immunotherapy following disease progression. For the MMRd subgroup, the median PFS with atezolizumab was not estimable, while the placebo group had a median PFS of 6.9 months (95% CI, 6.3–10.1). Patients treated with atezolizumab experienced a 64% reduction in the risk of disease progression compared to those receiving placebo (HR 0.36, 95% CI 0.23–0.57; *p* = 0.0005).

## 4. Ongoing Trials Investigating Immunotherapy in First Line as Monotherapy

Building on the evidence from the previously presented randomized clinical trials, the impact of immunotherapy on survival in MMRd endometrial carcinomas is now well established, particularly in the context of advanced or recurrent disease [2]. In light of the significance of these studies, one of the potential next steps is to reduce the combined toxicities of immunotherapy and standard chemotherapy by utilizing immunotherapy as a standalone first-line treatment. In this context, two randomized clinical trials are currently focusing on investigating the use of ICIs as monotherapy, comparing them to the gold standard, in patients with advanced-stage or first recurrent MMRd endometrial carcinomas. Specifically, the GINECO-EN105b/ENGOT-en13, DOMENICA trial (NCT05201547) aims to compare dostarlimab to standard chemotherapy [29]; the study aims to de-escalate first-line treatment for patients with MMRd/MSI-H endometrial cancer by replacing carboplatin-paclitaxel with the PD-1 inhibitor dostarlimab, thereby reducing the associated toxicities. Similarly, in the KEYNOTE-C93/GOG-3064/ENGOT-en15 clinical trial (NCT05173987), women with advanced or recurrent endometrial carcinoma exhibiting MMRd who have not previously received systemic chemotherapy are treated with pembrolizumab 400 mg every 6 weeks for up to 18 cycles (approximately 2 years) and compared to a regimen of carboplatin and paclitaxel [30]. The study aims to evaluate the efficacy and safety of pembrolizumab as a first-line solo therapy. Table 3 summarizes the populations, treatments, and outcomes of these studies.

## 5. Combined Therapies

An intriguing aspect of immunotherapy in the treatment of endometrial carcinoma, which is the focus of several ongoing studies, is its integration with other treatment modalities. Combining ICIs with agents such as PARP (Poly (ADP-ribose) polymerase) inhibitors (PARPi), anti-angiogenic therapies, and targeted molecular inhibitors shows promise for enhancing antitumor efficacy and overcoming resistance mechanisms [31,32]. PARPi target and inhibit PARP enzymes, which are critical for single-strand DNA break repair via the base excision repair pathway. By inhibiting PARP, PARPi encourage the accumulation of single-strand breaks, which are eventually converted into double-strand breaks during DNA replication. For instance, PARP inhibitors have demonstrated efficacy in cancers with DNA repair deficiencies [33]. Preclinical studies have shown that PARP inhibitors can increase the neoantigen burden [34,35,36]. Additionally, the disruption of DNA repair mechanisms can generate small DNA fragments that activate the cyclic GMP-AMP synthase (cGAS) and stimulator of interferon genes (STING) pathway, resulting in enhanced recruitment of CD8-positive T cells [37,38]. On the other hand, we have previously highlighted the efficacy of immunotherapy in MMRd tumors and the ongoing efforts towards de-escalating treatment to monotherapy in this subgroup of ECs, aiming to avoid overlapping toxicities from different drug classes. Consequently, the current focus of combination therapies is primarily on MMRp tumors, which are not the target of this review. Nonetheless, we will consider key trials that include MMRd tumors, even if they are not the primary target (Table 4). Conversely, trials involving only MMRp tumors or those not stratifying based on the MMR status have been excluded.

In this regard, the previously mentioned DUO-E study, which evaluates the efficacy of ICIs combined with Olaparib, demonstrated comparable efficacy between the group receiving durvalumab alone and the group treated with durvalumab plus olaparib in MMRd patients, whereas the addition of olaparib provided greater benefit, which was specifically observed in the MMRp subgroup [27]. In the recently completed phase II EndoBARR trial (NCT03694262), the efficacy and safety of a platinum-free combination therapy of bevacizumab, atezolizumab, and rucaparib were evaluated in 30 women with previously treated recurrent and progressive endometrial carcinoma [39]. The authors reported that this combination demonstrated a clinically meaningful improvement in response with acceptable toxicity (grade 3 or 4 treatment-related adverse events occurred in 50% of the patients). Moreover, an enhanced response to therapy was observed in patients with MMRd tumors; long-term survival data are awaited. Regarding the combination of ICIs and PARP inhibitors, it is also necessary to mention the ongoing RUBY (part 2) trial (NCT03981796), which is evaluating the combination of dostarlimab and standard-of-care chemotherapy, followed by dostarlimab plus niraparib as maintenance therapy, compared to placebo plus chemotherapy followed by placebo, in adult patients with primary advanced or recurrent MMRp/MSS endometrial cancer [25].

Numerous studies have evaluated the combination of ICIs and anti-angiogenic drugs, with particular focus on the combination of lenvatinib and pembrolizumab in the treatment of EC. Anti-angiogenic drugs inhibit the formation of new blood vessels by targeting vascular endothelial growth factor (VEGF) pathways, which are essential for tumor vascularization. By blocking VEGF signaling, these agents reduce the blood supply to tumors, limiting oxygen and nutrient delivery, ultimately inhibiting tumor growth and progression. However, most of these trials focused on the MMRp group. Nonetheless, the KEYNOTE-775 trial demonstrated that a certain level of efficacy can also be observed in the MMRd group [40,41]. The efficacy was significant enough to prompt the FDA to grant breakthrough therapy designation to lenvatinib in combination with pembrolizumab for the potential treatment of patients with recurrent/metastatic MMRp endometrial cancer that has progressed after at least one prior systemic therapy [42]. Regarding this combination, the phase III ENGOT-En9/LEAP-001 trial (NCT03884101) aimed to explore a chemotherapy-free treatment strategy for EC patients (stratified by their MMR status) [43]. This study evaluated the safety and efficacy of first-line pembrolizumab plus lenvatinib compared to the standard regimen of paclitaxel and carboplatin in newly diagnosed stage III/IV or recurrent endometrial cancer. The study did not meet the pre-specified statistical thresholds for either overall survival or progression-free survival in the MMRp group. However, it remains unclear how much benefit is achieved by adding lenvatinib to pembrolizumab in MMRd tumors and whether the potential increase in toxicity is justified in this patient group. The complete and detailed results are still pending publication. Another anti-angiogenic agent studied in combination with ICIs is anlotinib (a tyrosine kinase inhibitor, TKI), which was evaluated in a Chinese study by Wei et al. in combination with sintilimab (an anti-PD-1 monoclonal antibody) [44]. The primary objective of the study was to assess this drug combination in patients with endometrial cancer, regardless of the MMR status, who had progressed after platinum-based chemotherapy. However, the post hoc analyses revealed that the objective response rate (ORR) in MSI-H/MMRd patients was significantly higher compared to those without these markers (100% vs. 57.1%, *p* = 0.048). Furthermore, MSI-H/MMRd status was associated with prolonged PFS (HR = 2.96; 95% CI, 1.04 to 8.40; *p* = 0.04) compared to that of MSS/MMRp patients (PMID: 35623659). A phase II trial (NCT03526432) evaluated the combination of Atezolizumab and Bevacizumab in recurrent endometrial cancer patients, stratified by MMR status. The results showed a modest ORR with the combination; 13% of patients were MMRd. The author concluded their interim analysis, stating that the ORR for this combination approximates that seen with Lenvatinib/Pembrolizumab with far fewer side effects.

Another potential therapeutic strategy is represented by the so-called “Dual ICIs therapy”, which involves combining two immunotherapeutic agents: one targeting the PD-1/PD-L1 pathway and the other acting on CTLA-4. This approach is based on a 2016 study published in *Lancet Oncology* on melanoma, where the combination of nivolumab and ipilimumab was approved for the treatment of advanced melanoma after demonstrating superior survival outcomes compared to ipilimumab monotherapy (63.5% vs. 53.6% 2-year OS, respectively) [45]. However, the combination was associated with a significantly higher rate of grade 3/4 treatment-related adverse events (55% vs. 20%). A phase II randomized trial in patients with recurrent endometrial cancer previously treated with platinum-based chemotherapy evaluated durvalumab alone or in combination with tremelimumab (an anti-CTLA-4 agent) [46]. The results showed a limited objective response rate (ORR: 14.8% vs. 11.2%) and progression-free survival (PFS: 7.6 vs. 8.1 weeks), without significant differences between the two arms. However, the study population predominantly consisted of MMRp ECs. In contrast, NRG-GY025 phase II randomized trial (NCT05112601) evaluating nivolumab with or without ipilimumab is currently ongoing in MMRd recurrent EC patients, and it may potentially yield better outcomes [47]. Recently, a study was published evaluating the intraperitoneal administration of ipilimumab and nivolumab in patients with recurrent gynecologic malignancies with peritoneal carcinomatosis [48]. This study included only two cases of endometrial cancer, making it difficult to draw specific conclusions regarding this cancer type or the MMRd subclass in particular. However, it may serve as a foundation for future research.

**Table 4:** Ongoing clinical trials focused on immunotherapy combined with other drugs.

Trial Name (NCT ID)	Study Design	Drugs	Drug Class	Population	Treatment	Primary Endpoint	Estimated Study Completion and Status
**ENGOT-en9/** **LEAP-001 trial** [43] (NCT03884101)	Phase III RCT	Pembrolizumab (MK3475) Lenvatinib (E7080)	TKI anti-VEGF	Newly diagnosed stage III/IV or first recurrent endometrial cancer (stratified for MMR status)	Experimental: Lenvatinib daily and Pembrolizumab once at the start of each 3-week treatment cycle; active comparator: Paclitaxel + Carboplatin	PFS, OS	01/2025 Active, not recruiting
**ATAPEMBRO** **trial** [49] (NCT04014530)	Phase I/II	Ataluren (DB05016); Pembrolizumab (MK3475)	DMD drug, binding ribosomes	Metastatic MMRd EC (and MMRd and MMRp colorectal adenocarcinoma)	Phase I: 200 mg i.v. Pembrolizumab q3w and dose escalation of Ataluren. Phase II: 200 mg i.v. pembrolizumab q3w and Ataluren at MTD (maximum tolerated dose)	Toxicities and side effects	3/2023 Recruiting
**ABILITY-1 trial** [50] (NCT05086692)	Phase I/II	MDNA11 (C187359); Pembrolizumab (MK3475)	rIL-2	Advanced solid tumors (MSI-H EC)	Intervention I: MDNA11 i.v. q2w dose escalation until mRDE. Intervention II: MDNA11 + Pembrolizumab until cRDE	Tolerability; TRAEs; TEAEs	12/2026 Recruiting
**EndoBARR trial** [39] (NCT03694262)	Phase II	Bevacizumab (J9035); Atezolizumab (MPDL-3280A); Rucaparib (L01XK03)	Anti-VEGF; PARPi	Patients must have recurrent or persistent/progressive endometrial carcinoma, which is refractory to curative therapy or established treatments	Atezolizumab i.v. 1200 mg, first day of 21-day cycle; Bevacizumab i.v. 15 mg/kg, first day of 21-day cycle and Rucaparib 600 mg orally twice daily by continuous dosing	ORR	04/2023Completed
[51] (NCT03526432)	Phase II	Atezolizumab (MPDL-3280A); Bevacizumab (J9035)	Anti-VEGF	Advanced, recurrent, or persistent ECs that have relapsed or are refractory to established treatments	Bevacizumab i.v. 15 mg/kg, first day of 21-day cycle and Atezolizumab i.v. 1200 mg, first day of 21-day cycle	ORR	05/2025Active, not recruiting
**NRG-GY025** [47] (NCT05112601)	Phase II	Nivolumab(MDX-1106) Ipilimumab (MDX-010)	Anti-CTLA4	Recurrent MMRd ECs	Arm I: Nivolumab i.v. on day 1 of each cycle and Ipilimumab i.v. on day 1 of every other cycle, q3w for up to 8 cycles (in the absence of disease progression, unacceptable toxicity, or CR). Arm II: Nivolumab	PFS	04/2026 Recruiting
[52] (NCT03367741)	Phase II	Cabozantinib S-malate (XL184); Nivolumab (MDX-1: 106)	TKI	Advanced, recurrent, or metastatic endometrial cancer previously treated with at least one line of platinum-based chemotherapy	Arm I: Cabozantinib S-malate orally on days 1–28 and Nivolumab i.v. on days 1 and 15 Arm II: Nivolumab	PFS	12/2024 Active, not recruiting

NCT: national clinical trial; RCT: randomized clinical trial; anti-VEGF: anti-vascular epithelial growth factor; EC: endometrial cancer; MMRd: mismatch repair-deficient; MMRp: mismatch repair-proficient; ORR: objective response rate; anti-CTLA4: anti-cytotoxic T-lymphocyte antigen 4; PFS: progression-free survival; OS: overall survival; TKI: tyrosine kinase inhibitor, DMD: Duchenne muscular dystrophy; q3w: every 3 weeks; q2w: every 2 weeks; rIL-2: recombinant interleukin 2; MSI-H: microsatellite instability-high; TRAE: treatment adverse event; PARPi: poly(ADP-ribose) polymerase inhibitor; CR: complete response.

### Immunotherapy Combination with Novel Therapies

The efficacy of ICIs in endometrial cancer, particularly in MMRd tumors, has driven research toward combining immunotherapy with novel drug classes or repurposing agents used for the treatment of other diseases, both oncological and non-oncological. Table 4 summarizes the characteristics of the studies in question.

The ATAPEMBRO trial (NCT04014530) is an open-label, single-center, Phase I/II trial aimed at evaluating the safety and efficacy of combining Ataluren with Pembrolizumab for the treatment of metastatic MMRd EC, as well as MMRd and MMRp colorectal adenocarcinoma [50]. Ataluren is a drug designed to bypass premature stop codon mutations in DNA by binding to the ribosome during mRNA translation, previously used to treat genetic disorders (e.g., Duchenne muscular dystrophy). This binding helps the ribosome ignore the premature stop codon (PTC) and continue protein synthesis, allowing for the translation of additional out-of-frame sequences that are abundant in MMRd tumors. According to the authors, this could lead to the generation of new target peptides for the immune system, potentially enhancing the effectiveness of Pembrolizumab. The primary objectives of the study are to assess the safety and tolerability of the combined treatment and the objective response rate as measured by immune-related response criteria. Secondary objectives include the immune-related progression-free survival, overall survival, progression-free survival, and overall response rate.

The ABILITY-1 trial (NCT05086692) encompasses patients with various advanced solid tumors, including advanced endometrial cancer [51]. This study is a Phase 1/2, open-label, dose-escalation, and expansion trial designed to assess the safety, tolerability, pharmacokinetics, pharmacodynamics, and preliminary antitumor activity of MDNA11. MDNA11 is a long-acting “beta-only” recombinant interleukin-2 (IL-2) superkine engineered to activate immune effector cells, such as CD8+ T cells and NK cells, which target cancer cells while minimizing the activation of immunosuppressive regulatory T cells. The study comprises two main phases: monotherapy, where MDNA11 is administered alone, and combination therapy, where MDNA11 is combined with pembrolizumab. Early results from the dose-escalation phase demonstrated promising outcomes, including durable tumor control and a manageable safety profile. The dose-expansion phase is currently ongoing, with a focus on specific cancer types that may derive the most benefit from MDNA11, such as melanoma and tumors characterized by MSI-H/MMRd, such as endometrial carcinoma.

The AFT-50 EndoMAP trial (NCT04486352) is a Phase IB/II multi-cohort clinical trial designed to evaluate the efficacy and safety of targeted agents with or without immunotherapy (atezolizumab) in patients with recurrent or persistent endometrial cancer [53]. This trial utilizes a biomarker-driven approach, grouping participants into cohorts based on the specific genomic profile of their tumors, using the FoundationOne CDx (F1CDx) NGS assay for genomic tumor profiling. The goal is to match each patient to the most appropriate targeted therapy or combination regimen. Patients with MSI-H tumors will receive a dual therapy of atezolizumab and tiragolumab. Tiragolumab is a monoclonal antibody specifically targeting TIGIT (T cell immunoreceptor with Ig and immunoreceptor tyrosine-based inhibitory motif domains), a co-inhibitory receptor expressed on various immune cells. In several solid malignancies, TIGIT is notably upregulated in T cells and NK cells [54]. Together with its ligand, poliovirus receptor-related 2 (PVRL2), which is also highly expressed in endometrial cancer, TIGIT forms a key pathway contributing to immune evasion. By inhibiting TIGIT, tiragolumab can potentiate the effects of anti-PD-1/PD-L1 therapies, resulting in enhanced clinical outcomes such as higher objective response rates (ORRs) and longer progression-free survival (PFS).

One last trial is NCT03367741, a randomized phase II study evaluating the efficacy and safety of combining cabozantinib, a TKI, with nivolumab in patients with advanced, recurrent, or metastatic endometrial cancer [52]. The trial is designed to compare two arms: Arm A (cabozantinib plus nivolumab) and Arm B (nivolumab monotherapy). Eligible patients include those who have progressed following at least one line of platinum-based chemotherapy, regardless of their microsatellite instability (MSI) or mismatch repair (MMR) status. The primary endpoint is progression-free survival (PFS).

## 6. Future Applications: Early-Stage Disease Setting

The proven efficacy of immunotherapy in treating advanced and recurrent MMRd endometrial carcinoma has led to the development of new trials aimed at expanding its application beyond the current standard settings. Traditionally used in the management of advanced-stage disease and first recurrences, ICIs are now being explored in the treatment of early-stage endometrial cancer, particularly in patients with MMRd status. This shift aims to harness the benefits of immunotherapy earlier in the treatment continuum, potentially reducing the risk of relapse and the need for chemoradiotherapy. Consequently, ongoing trials are assessing the potential of ICIs in adjuvant and neoadjuvant contexts, as well as their integration into fertility-sparing protocols for select patient populations (Table 5). The ultimate goal is to develop strategies that allow the use of immunotherapy as a standalone treatment option or in combination with other modalities, thereby minimizing long-term treatment-related morbidities while maintaining oncologic safety. In this context, a new study, the ENGOT-en11/GOG-3053/KEYNOTE-B21 study, has been recently presented, further contributing to our understanding of pembrolizumab’s efficacy in the high-risk MMRd EC population [55]. The ENGOT-en11 trial is a phase 3 randomized study that evaluates the effectiveness of pembrolizumab plus adjuvant chemotherapy, with or without radiotherapy, in patients with newly diagnosed, stage I-II high-risk EC following surgery with curative intent. In this double-blind trial, 1095 patients were randomized to receive pembrolizumab or a placebo, both in combination with carboplatin–paclitaxel chemotherapy. At the time of the interim analysis, no significant difference in DFS was observed between the pembrolizumab and placebo groups (hazard ratio 1.02, *p* = 0.57), with two-year DFS rates of 75% and 76%, respectively. However, a subgroup analysis revealed that in the mismatch repair-deficient (MMRd) group, pembrolizumab significantly improved the DFS (HR 0.31). No survival benefit was observed in the mismatch repair-proficient (MMRp) population.

Some ongoing trials are currently investigating various aspects of immunotherapy use in early-stage EC, by exploring the efficacy of ICIs in different therapeutic windows: the RAINBO (Refining Adjuvant Treatment Based on Molecular Features) clinical trial program is evaluating the combination of pembrolizumab with standard therapies for high-risk MMRd endometrial cancer patients [56]. Early-phase studies have shown that neoadjuvant pembrolizumab can result in significant tumor regression, potentially improving surgical outcomes and reducing recurrence rates. The RAINBO program is distinguished by its personalized treatment approach, which tailors adjuvant therapies based on specific molecular characteristics. The program includes several parallel clinical trials: MMRd-GREEN evaluates the efficacy of adjuvant anti-PD-L1 inhibitors in stage II-III MMRd endometrial carcinoma (EC) with significant lymphovascular space invasion (LVSI); p53abn-RED assesses the effectiveness of a 2-year maintenance therapy with niraparib following chemoradiotherapy in stage I-III p53abn EC; NSMP-ORANGE compares the addition of hormone therapy to external beam radiation therapy (EBRT) versus the addition of chemotherapy to EBRT in stage II-III NSMP EC patients; and POLEmut-BLUE, where patients with POLEmut EC undergo follow-up only, without adjuvant treatment, except for advanced stages that receive EBRT. This program aims to establish new standards in personalized cancer therapy by offering more effective and less toxic treatment options.

**Table 5:** Ongoing clinical trials focused on immunotherapy in early-stage MMRd ECs.

Trial Name (NCT ID)	Study Design	Drugs	Population	Treatment	Primary Endpoint	Estimated Study Completion and Status
**NRG-GY020 trial** [57] (NCT04214067)	Phase III RCT	Pembrolizumab (MK3475)	High-/intermediate-risk stage I/II MMRd EC	Experimental arm II: EBRT, VBRT + pembrolizumab IV within 7 days prior to the start of radiation therapy. Treatment with pembrolizumab repeats every 6 weeks for 9 cycles. Control arm: EBRT + VBRT.	3-year RFS	2/2025 Recruiting
**RAINBO** **MMRd-GREEN trial GCIG/DGOG/ENGOT-EN142** (NCT05255653)	Phase III RCT	Durvalumab	Stage III EC (MMRd) or stage IB/II EC with substantial LVSI	Experimental arm: adjuvant radiotherapy (45.0–48.6 Gy; 1.8–2.0 Gy per fraction, 5 fractions a week) combined with and followed by durvalumab, 1500 mg intravenous once every 4 weeks for, in total, 1 year (13 cycles) starting in the first week of radiotherapy. Control arm: adjuvant pelvic EBRT (45.0–48.6 Gy; 1.8–2.0 Gy per fraction, 5 fractions a week).	RFS	01/2031 Recruiting
**SATELLITE trial** (NCT06278857)	Phase IIb	Dostarlimab (TSR-042)	Stage I, FIGO G1-2, MMRd, endometrioid EC, and wish to preserve the uterus or not a suitable candidate for hysterectomy.	Dostarlimab 4 cycles q3w, 34w rest period, 3 cycles q6w (total 7 cycles).	pCR	01/06/2028 Not yet recruiting

NCT: national clinical trial; RCT: randomized clinical trial; EC: endometrial cancer; MMRd: mismatch repair-deficient; EBRT: external beam radiotherapy; VBRT: vaginal brachytherapy; RFS: recurrence-free survival; LVSI: lymphovascular space invasion; Gy: Gray; q3w: every 3 weeks; q6w: every 6 weeks; pCR: pathological complete response.

Another ongoing trial focused on adjuvant treatment, the NRG-GY020 (NCT04214067), aims to evaluate the efficacy of combining radiation therapy with pembrolizumab in patients newly diagnosed with stage I–II high-/intermediate-risk endometrioid endometrial cancer MMRd [57]. In this study, participants are randomized into two groups: Arm I: patients receive standard pelvic external beam radiation therapy (EBRT) followed by vaginal brachytherapy. Arm II: patients receive the same radiation treatment as Arm I, with the addition of pembrolizumab administered intravenously every six weeks for up to one year (a total of nine cycles), beginning within seven days prior to the start of radiation therapy. The primary objective of the trial is to compare three-year progression-free survival (PFS) between the two groups, determining whether the addition of pembrolizumab enhances outcomes for this specific patient population.

Immunotherapy may also have potential applications in the treatment of early-stage endometrial cancer as an alternative to surgery. The SATELLITE Study (feaSibility sAfeTy Efficacy dostarLimab earLy-stage defIcient endomeTrial cancEr) (NCT06278857) is designed to evaluate the efficacy and safety of dostarlimab in early-stage endometrial cancer [58]. This study explores the potential of dostarlimab as a non-surgical option for patients who are either unsuitable or unwilling to undergo major surgery, allowing for fertility preservation or addressing specific health concerns. Specifically, the trial aims to enroll patients with histologically or cytologically confirmed Stage 1, FIGO grade 1 or 2, MMRd endometrioid EC who wish to preserve their uterus (organ-sparing approach) or are not suitable candidates for hysterectomy. The primary objective of the study is to determine the absence of endometrial cancer following the dostarlimab treatment regimen. The authors will also assess the safety and tolerability of dostarlimab in participants with early-stage MMR-deficient endometrioid endometrial adenocarcinoma.

Within the context of an organ-sparing approach, a recent study published in *Nature Communications* explored the use of neoadjuvant pembrolizumab therapy in patients with resectable MMRd endometrial carcinoma (80% FIGO stage I, 10% II, 10% III) [59]. This phase I study included 10 women treated with two cycles of pembrolizumab (200 mg administered intravenously every three weeks) prior to their planned surgical intervention. The primary endpoint was pathological response, while secondary endpoints included the radiological response and safety profile. The results revealed a pathological response in 50% of the patients (two cases with a major pathological response and three with a partial response), along with a partial radiological response in 37.5%. All of the patients completed the treatment without severe toxicity. During the median follow-up period of 22.5 months, two recurrences were observed among the non-responders. Although no complete pathological responses were achieved, the safety and preliminary efficacy data seem to support the further exploration of neoadjuvant ICIs in this context, with the potential to expand the organ-sparing approach for MMRd endometrial cancer, especially in young patients with childbearing desire who are affected by hereditary MMRd-associated cancers, typical of Lynch syndrome.

## 7. Conclusions

This review outlines the pivotal role of immunotherapy in the evolving treatment paradigm for mismatch repair-deficient (MMRd) endometrial cancer, particularly in the context of advanced and recurrent disease. While ICIs have shown significant clinical efficacy, especially in MMRd/MSI-H tumors, the integration of these agents into therapeutic regimens remains a complex and nuanced process. This article underscores the potential of combining ICIs with other treatment modalities such as chemotherapy, targeted therapies (e.g., PARP inhibitors), and radiotherapy to enhance therapeutic efficacy and overcome resistance. Current evidence from randomized clinical trials supports the use of pembrolizumab, dostarlimab, and other ICIs as a first-line option in patients with MMRd endometrial carcinoma who have progressed after platinum-based chemotherapy. However, the conclusive data from major phase III trials will be pivotal for establishing the true long-term efficacy of immunotherapy in first-line treatment settings and for shaping future strategies for advanced disease management.

Future research should focus on identifying specific biomarkers that can predict the response to immunotherapy and on establishing optimal combination strategies tailored to different molecular subtypes. Additionally, the role of ICIs in the adjuvant and neoadjuvant settings remains to be fully defined. While preliminary results from ongoing trials are promising, it is crucial to continue exploring innovative therapeutic combinations that target both the primary tumor and metastatic disease, thereby reducing recurrence rates and improving long-term outcomes. The review also highlights the need for a multidisciplinary approach to integrate emerging molecular diagnostics with clinical practice to optimize treatment decisions and ensure that patients receive the most effective personalized care possible. In conclusion, immunotherapy has marked a significant advancement in the treatment of MMRd endometrial cancer, and continued research is essential to maximize its potential and establish new standards of care in this heterogeneous disease.

## Figures and Tables

**Table 1:** Main randomized controlled (RC) studies investigating immunotherapy in MMRd endometrial cancer.

Trial Name (NCT ID)	Author	Design of the Study	Population	Treatment		Maintenance		Primary Endpoint	Secondary Endpoint
				Experimental Arm	Control Arm	Experimental Arm	Control Arm		
**MITO END-3 trial (2024)** [24] (NCT03503786)	Pignata, S., et al.	RC phase II	FIGO stage III and IV or recurrent EC with no previous systemic CHT as primary treatment for advanced or metastatic disease	Carboplatin and Paclitaxel + Avelumab every 3 weeks for 6 cycles	Carboplatin and Paclitaxel + Placebo every 3 weeks for 6 to 8 cycles	Avelumab every 2 weeks	Placebo	PFS (in the ITT population)	OS; QoL; objective response rate; duration of response; safety and tolerability
**RUBY trial (2023)** [25] (NCT03981796)	Mirza, M.R., et al.	RC phase III	Primary treatment for advanced or metastatic disease (FIGO stage III or IV) or metastatic disease not amenable to curative therapy	Carboplatin and Paclitaxel + Dostarlimab every 3 weeks for 6 cycles	Carboplatin and Paclitaxel + Placebo every 3 weeks for 6 cycles	Dostarlimab every 6 weeks	Placebo every 6 weeks	PFS; OS (in the overall population)	Objective response; safety; disease control; response duration; time to second PD; PROs; pharmacokinetic and immunogenicity analyses
**NRG GY018 trial (2023)** [26] (NCT03914612)	Eskander, R.N., et al.	RC phase III	Advanced (FIGO stage III-IV) or recurrent EC of any histologic subtype (except for carcinosarcoma)	Carboplatin and Paclitaxel + Pembrolizumab for 6 cycles	Carboplatin and Paclitaxel + Placebo for 6 cycles	Pembrolizumab every 6 weeks for 14 cycles	Placebo every 6 weeks for 14 cycles	PFS (in two cohorts based on MMR status)	Safety; OS; QoL
**DUO-E trial (2024)** [27] (NCT04269200)	Westin, S.N., et al.	RC phase III	Newly diagnosed advanced (FIGO stage III-IV) or recurrent EC of epithelial histology (excluding sarcomas)	2 experimental arms: both receiving Carboplatin and Paclitaxel + Durvalumab every 3 weeks for 6 cycles	Carboplatin and Paclitaxel + Placebo every 3 weeks for 6 cycles	2 arms: (i) Durvalumab every 4 weeks; (ii) Durvalumab and Olaparaib every 4 weeks	Placebo every 4 weeks	PFS (in the ITT population)	OS; QoL; safety
**AtTEnd trial (2024)** [28] (NCT03603184)	Colombo, N., et al.	RC phase III	EC with residual disease after surgery, or inoperable FIGO stage III–IV EC or carcinosarcoma with no previous systemic anticancer therapy, or recurrent disease if not previously treated with chemotherapy for recurrence	Carboplatin and Paclitaxel + Atezolizumab every 3 weeks for 6–8 cycles	Carboplatin and Paclitaxel + Placebo every 3 weeks for 6–8 cycles	Atezolizumab every 21 days	Placebo every 21 days	PFS, OS (in the ITT population)	Objective response rate; duration of response; second PFS; QoL; safety; pharmacokinetics; anti-drug antibodies

RC: randomized controlled; EC: endometrial cancer; CHT: chemotherapy; PFS: progression-free survival; ITT: intention to treat; OS: overall survival; QoL: Quality of Life; PD: progressive disease; PROs: patient-reported outcomes.

**Table 2:** Key characteristics of MMRd samples in major randomized trials investigating the use of immunotherapy in advanced or first recurrence ECs.

	**MITO END-3 Trial [24]** (NCT03503786)		**RUBY Trial** [25] (NCT03981796)		**NRG GY018 Trial** [26] (NCT03914612)		**DUO-E Trial** [27] (NCT04269200)			**AtTEnd Trial** [28] (NCT03603184)	
	Experimental Arm	Control Arm	Experimental Arm	Control Arm	Experimental Arm	Control Arm	Experimental Arm 1	Experimental Arm 2	Control Arm	Experimental Arm	Control Arm
**Histology**, n (%)											
Endometrioid	44 (70)	46 (74)	44 (83) ^§^	56 (86) ^§^	88 (78.5) ^§^	92 (81.4) ^§^	141 (59.2)	152 (63.6)	139 (57.7)	74 (95) ^§^	38 (88) ^§^
Serous	10 (16)	9 (15)	1 (2) ^§^	1 (2) ^§^	4 (3.6) ^§^	1 (0.9) ^§^	58 (24.4)	42 (17.6)	54 (22.4)	0 ^§^	0 ^§^
Clear cells	3 (5)	1 (2)	0 ^§^	0 ^§^	1 (0.9) ^§^	0 ^§^	4 (1.7)	8 (3.3)	7 (2.9)	0 ^§^	0 ^§^
Carcinosarcoma	0	0	4 (8) ^§^	1 (2) ^§^	Excluded	Excluded	12 (5)	18 (75)	21 (8.7)	3 (4) ^§^	1 (2) ^§^
Undifferentiated	4 (6)	3 (5)	0 ^§^	0 ^§^	4 (3.6) ^§^	4 (3.5) ^§^	4 (1.7)	5 (2.1)	3 (1.2)	1 (1) ^§^	3 (7) ^§^
Mucinous	1 (2)	0	0 ^§^	0 ^§^	0 ^§^	0 ^§^	1 (0.4)	0	0	0 ^§^	0 ^§^
Mixed	1 (2)	3 (5)	2 (4) ^§^	4 (8) ^§^	3 (2.7) ^§^	2 (1.8) ^§^	9 (3.8)	9 (3.8)	11 (4.6)	3 (4) ^§^	1 (2) ^§^
Other	0	0	2 (4) ^§^	3 (5) ^§^	12 (10.7) ^§^	14 (12.4) ^§^	9 (3.8%)	5 (2.1)	6 (2.5)	0 ^§^	1 (2) ^§^
**FIGO stage at diagnosis (2009)**, n (%)											
I-II	20 (32)	24 (39)	21 (40) ^§^	27 (42) ^§^	-	-	0	1 (0.9)	1 (0.4)	39 (49) ^§^	18 (41) ^§^
III	23 (37)	22 (35)	14 (26) ^§^	20 (31) ^§^	-	-	17 (7.1)	12 (5.0)	12 (5.0)	16 (20) ^§^	7 (16) ^§^
IV	20 (32)	16 (26)	14 (26) ^§^	15 (23) ^§^	-	-	96 (40.3)	99 (41.4)	101 (41.9)	26 (32) ^§^	19 (43) ^§^
Unknown	0	0	4 (8) ^§^	3 (5) ^§^	-	-	-	-	-	0 ^§^	0 ^§^
**Status of the disease at study** **entry in MMRd pop**, n (%)											
New diagnosis FIGO stage III-IV	30 (48)	30 (48)	26 (49)	33 (51)	-	-	113 (47.5)	114 (47.7)	115 (47.7)	29 (36)	16 (36)
Recurrence	33 (52)	32 (52)	27 (51)	32 (49)	-	-	125 (52.5)	125 (52.3)	126 (52.3)	52 (64)	28 (64)
**Population MMRd**, n (%)	31	26	53 (21.6)	65 (26.1)	112 (27.7)	113 (27.7)	46 (19.3)	48 (20.1)	49 (20.3)	81 (22.5)	44 (23)
**Previous chemotherapy in MMRd pop**, n (%)	-	-	7 (13.2)	10 (15.4)	5 (4.5)	8 (7.1)	-	-	-	14 (17)	11 (25)
**Previous radiotherapy**	29 (46%)	28 (45%)	8 (15)	13 (20)	155 (38.3)	174 (42.7)	73 (30.7)	85 (35.6)	71 (29.5)	-	-
**Mean follow-up**, month	23.3	23.5	24.8	24.8	12	12	15.4	15.4	12.6	28.3	28.3
**Objective response rate**	17%	6%	77.6%	69%	81.5%	70.7%	-	-	-	82.4%	75.7%
**Complete response rate**	41 (65%)	31 (50%)	53 (25.0)	43 (19.6)	-	-	-	-	-	-	-
**PFS in MMRd patients**, months—median (95% CI)	8.0 (4.8–12.3)	NE (8.9–NE)	NE (12.4–NE)	6.9 (6.3–10.1)	NE (30.6–NE)	NE (30.6–NE)	NE (NE-NE)	31.8 (12.4-NE)	7.0( 6.7–14.8)	NE (12.4–NE)	6.9 (6.3–10.1)
**PFS in MMRd**, HR (95% CI)	0.46 (0.22–0.94)		0.28 (0.16–0.50)		0.30 (0.19–0.48)		0.41 (0.21–0.75) *; 0.42 (0.22–0.80) **; 0.97 (0.49–1.98) ***			0.36 (0.23–0.57)	
**OS in MMRd**, months—median (95% CI)	NE (NE–NE)	26.1 (13.0–NE)	83.3 (66.8–92.0)	58.7 (43.4–71.2)	-	-	-	-	-	NE (NE–NE)	25.7(14.5–NE)
**OS in MMRd**, HR (95% CI)	0.41 (0.14–1.18)		0.30 (0.13–0.70)		-		-			0.41 (0.22–0.76)	
**Adverse events in overall population (Clavien–Dindo classification)**, n (%)	455	361	241	246	365	361	232	237	236	351	185
Grade 1–2	350	289	-	-	144	187	103	77	103	113 (32)	67 (36)
Grade 3	84	59	-	-	214	170	129	160	133	157 (44)	86 (46)
Grade 4	19	13	-	-						70 (20)	28 (15)
Grade 5	2	0	-	-	7	4				11 (3)	4 (2)

^§^ Data specifically related to the MMRd population. * Durvalumab + olaparib vs. control. ** Durvalumab vs. control. *** Durvalumab + olaparib vs. durvalumab. NE: not estimable. -: not reported. NCT: national clinical trial; MMRd: mismatch repair-deficient; PFS: progression-free survival; OS: overall survival; HR: hazard ratio.

**Table 3:** Ongoing clinical trials focused on immunotherapy as monotherapy.

Trial Name (NCT ID)	Study Design and Status	Drugs	Population	Treatment	Primary Endpoint	Estimated Study Completion and Status
**DOMENICA GINECO-EN105b/ENGOT-en13** [29] (NCT05201547)	RCT phase III	Dostarlimab (TSR-042)	First recurrent or primary advanced (stage III-IV) EC	Experimental arm: Dostarlimab 500 mg, every 3 weeks, four cycles and then 1000 mg every 6 weeks. Control arm: Carboplatin AUC 5 or 6 plus Paclitaxel 175 mg/m^2^, every 3 weeks, six cycles.	PFS	10/2029 Recruiting
**KEYNOTE-C93/** **MK-3475-C93/ GOG-3064/** **ENGOT-en15** [30] (NCT05173987)	RCT phase III	Pembrolizumab (MK3475)	Advanced or recurrent MMRd EC not previously treated with chemotherapy	Experimental arm: Pembrolizumab 400 mg i.v. every 6 weeks (q6w) for up to 18 cycles (up to approximately 2 years). Control arm: paclitaxel 175 mg/m^2^ and carboplatin AUC 5 or 6 every 3 weeks (q3w) for 6 cycles (up to approximately 4 months).	PFS, OS	5/2027 Active, not recruiting

NCT: national clinical trial; RCT: randomized controlled trial; EC: endometrial cancer; MMRd: mismatch repair-deficient, PFS: progression-free survival; OS: overall survival; AUC: area under the curve; q6w: every 6 weeks; q3w: every 3 weeks.

## Data Availability

The data presented in this study are available upon request from the authors.
